# Corticobasal degeneration and corticobasal syndrome: A review

**DOI:** 10.1016/j.prdoa.2019.08.005

**Published:** 2019-08-30

**Authors:** Vasilios C. Constantinides, George P. Paraskevas, Panagiotis G. Paraskevas, Leonidas Stefanis, Elisabeth Kapaki

**Affiliations:** a 1st Department of Neurology, National and Kapodistrian University of Athens, School of Medicine, Eginition Hospital, Greece; b Department of Nursing, Technological Educational Institute of Crete, School of Health and Welfare Services, Greece

**Keywords:** Corticobasal syndrome, Corticobasal degeneration, Biomarkers, MRI, PET, Spect, Cerebrospinal fluid

## Abstract

Corticobasal degeneration (CBD) is a rare neurodegenerative disorder. The most common presentation of CBD is the corticobasal syndrome (CBS), which is a constellation of cortical and extrapyramidal symptoms and signs. Clinical-pathological studies have illustrated that CBD can present with diverse clinical phenotypes, including a non-fluent, agrammatic primary progressive aphasia syndrome, a behavioral, dysexecutive and visuospatial syndrome, as well as a progressive supranuclear palsy-like syndrome. Conversely, multiple pathologies, such as CBD, Alzheimer's disease and progressive supranuclear palsy may underlie a patient with CBS. This clinical-pathological overlap emphasizes the need for biomarkers that will assist in the accurate diagnosis of patients with CBS. This review presents an overview of the pathological, genetic, clinical and therapeutic characteristics of CBD, with an emphasis on the imaging (structural and functional) and biochemical (cerebrospinal fluid) biomarkers of CBD.

## 1 Introduction

Rebeiz et al. first described in 1967 three patients with slowly progressive “clumsiness” and a variety of asymmetrical extrapyramidal signs, including rigidity, dystonia and myoclonus, with relatively intact cognitive functions [1]. They introduced the term “corticonigral degeneration with neuronal achromasia” to describe this entity. This disorder was largely forgotten until 1989, when Marsden et al. introduced the term “corticobasal degeneration” [2].

Over the following years, the terms corticobasal degeneration (CBD), which refers to the pathological entity of a specific 4-repeat (4R) tauopathy, and corticobasal syndrome (CBS), which refers to the phenotype, have been used interchangeably. This has greatly added to the confusion surrounding the subject, implying that all CBD patients present with a CBS phenotype and vice versa. Pathological criteria were established in 2002 [3], and clinical diagnostic criteria in 2013 [4], further adding to the uncertainty.

Clinical-pathological studies have greatly enhanced our knowledge of the biochemistry, genetics, pathology and clinical manifestations of this rare neurodegenerative disorder. These studies have illustrated the clinical diversity of CBD, which can present with the classical CBS phenotype, but also as primary progressive aphasia, a frontal-dysexecutive-spatial syndrome and a Richardson-like syndrome [4]. Conversely, the pathological heterogeneity of CBS also has been emphasized, with diverse diseases such as CBD, Alzheimer's disease (AD), progressive supranuclear palsy (PSP) and frontotemporal degeneration with TDP-43 (FTD-TDP43) manifesting as CBS [5].

## 2 Epidemiology

Robust epidemiological data on CBD are lacking because of the rarity of the disease, the great clinical pathological heterogeneity of the disorder and the lack of established criteria until recently. It is estimated that it is ten times rarer than progressive supranuclear palsy (PSP) [6]. Its' incidence is estimated at 0.6–0.9/100,000/year and it represents 4–6% of patients with Parkinsonism. A single study projected a prevalence of 4.9–7.3 per 100.000, based on incidence and mean survival data. However, in a cohort of 120,000, no case of CBD was described [7,8].

Mean age at disease onset is about 64 years [4], whereas the youngest pathologically confirmed case was 43 years old [9]. There could be a slight female predominance [10,11]. Mean survival is estimated at 6.5 years [4].

## 3 Pathology

Dickson et al. established pathological criteria for CBD in 2002 [3]. CBD is characterized by neuronal and glial pathological lesions, which contain abnormally hyper-phosphorylated microtubule associated tau protein. For this reason, CBD is considered a tauopathy, alongside PSP, frontotemporal degeneration (FTD) and Alzheimer's disease.

Tau protein can present in six isoforms. This depends on the presence of three (3R) or four (4R) microtubule binding regions, due to alternate splicing of exon 10 of the microtubule associated protein tau (*MAPT*) gene, as well as the presence of no, one or two oligonucleotides (N1, N2), coded by exons 2 and 3 respectively in the N-terminal segment of tau protein.

CBD and PSP are 4R-tauopathies, FTD (Pick's disease) is a 3R-tauopathy and AD harbors both 3R- and 4R-tau isoforms. Differential diagnosis on a pathological basis of CBD from other tauopathies relies on specific pathological lesions as well as diverse distribution of these lesions among different diseases.

Ballooned or achromatic neurons were considered pathognomonic of CBD by Rebeiz et al., particularly when located in the cortical gray matter or less commonly in basal ganglia [1]. However, it is now evident that tau pathology in astrocytes is more important in discriminating PSP from CBD. Thus, astrocytic plaques are pathognomonic of CBD, whereas PSP is characterized by tufted astrocytes. Other, less specific tau-lesions of CBD are neuronal inclusions, threads and coiled bodies [12,13]. Coiled bodies, which are oligodendroglial tau depositions also differ between PSP and CBD, but on an ultra-structural level [14,15]. Moreover, CBD has more widespread cortical pathology, whereas PSP has more hindbrain pathology [16]. It is worth mentioning that even pathological examination cannot safely differentiate PSP from CBD (90% specificity) [3].

CBD appears to be a neural network specific disorder, as is the case with many other neurodegenerative diseases. It initially affects the dorsolateral prefrontal cortex and basal ganglia circuits, with more posterior regions affected as the disease progresses. Importantly, the initial pathological disorder is the astrocytic plaque, with neurons and oligodendroglia being affected in more advanced disease stages. Phenotypical variation in CBD depends on the topography and burden of the pathological lesions [17].

Interestingly, despite being 4-R tauopathies, PSP and CBD have some biochemical differences. As indicated by immunoblotting of brain extracts, diverse tau fragments are present in PSP and CBD, implying different tau proteolytic pathways. These differences may contribute to the phenotypical variation of the two disorders [18].

## 4 Diagnostic criteria

Various sets of diagnostic criteria have been proposed over the years, focusing exclusively on the CBS presentation of CBD [[19], [20], [21], [22], [23], [24], [25]]. The most recent established clinical diagnostic criteria, which were introduced in 2013, aimed to expand the phenotypical spectrum of CBD, by incorporating diverse clinical phenotypes [4]. These criteria were based on clinical-pathological studies of CBD and attempted to illustrate the great clinical–pathological diversity of CBS and CBD.

Based on these criteria, five clinical syndromes are accepted for CBD. These include the probable and possible CBS; a frontal-behavioral and spatial syndrome (FBS); a non-fluent agrammatic primary progressive aphasia syndrome (nfa-PPA); and a progressive supranuclear palsy-like syndrome (PSPS).

A probable CBD diagnosis requires an age of onset greater than 50 years, whereas a family history or a known genetic mutation affecting tau protein is not permitted. Of the five phenotypes, only the probable CBS and the FBS and nfa-PPA (with the addition of a CBS feature) are eligible for a probable CBD diagnosis.

For a possible CBS diagnosis, the criteria are more lenient. No age of disease onset is required, and a positive family history or a known tau-protein mutation is allowed. A possible CBD diagnosis can be established with a possible CBS phenotype, an FBS or nfa-PPA syndrome (without additional CBS features), or in cases of a PSP-S with an additional CBS feature.

Two studies examined the sensitivity and specificity of these clinical criteria, based on small cohorts of pathologically confirmed CBD patients [26,27]. Both studies concluded that the current diagnostic criteria lack specificity and that no clinical feature can confidently differentiate between CBD and a non-CBD pathology (more commonly AD or PSP) in a CBS patient. This emphasizes the need for biomarkers to determine the underlying pathology in a CBS patient.

## 5 Clinical features

Corticobasal syndrome is characterized by cortical and extrapyramidal signs. Apraxia, cortical sensory deficits and alien limb phenomena are the most common cortical signs, whereas asymmetrical Parkinsonism, dystonia and myoclonus comprise the extrapyramidal signs.

Parkinsonism in corticobasal syndrome is characteristically highly asymmetrical or unilateral. There have been rare reports of symmetric corticobasal degeneration [28]. In a minority of patients there can be moderate but transient levodopa response. Tremor, when present, is phenotypically atypical. It usually is a positional or action tremor, can be irregular and often has a myoclonic quality [4]. Typical levodopa-induced dyskinesias, resembling PD, have also been described [29].

Dystonia is present in 40% of CBD patients [30]. About 80% of patients have upper limb dystonia, whereas cervical, lower limb dystonia and blepharospasm are rare. Usually dystonia presents within the first two years of the disease course and is highly related to myoclonus.

Myoclonus seems to be of the cortical reflex type, based on giant somatosensory potentials and the latency between EEG and EMG [31]. Myoclonus in CBD has been hypothesized to result from abnormal hyper-excitability of the primary motor cortex, due to lack of inhibitory input from the sensory cortex [32]. The lack of a giant somatosensory potential in cases of CBD has been attributed to the profound parietal atrophy in the later stages of CBD [33].

Apraxia is the clinical hallmark of CBS. Although considered relatively specific for CBD, some apraxia studies have argued that apraxia is present in as many as 75% of PSP patients [34,35] and 30% of PD patients [36]. Quantitative analyses, however, have demonstrated that there are differences in the severity of apraxia (particularly in intransitive movements) [34,37]. Moreover, qualitative analyses, including error-type profiling, could also differentiate between the two disorders [35]. CBD patients have more severe distal than proximal apraxia, which supports the notion of limb-kinetic apraxia [38].

The alien-limb syndrome was initially described as a subjective difficulty recognizing one's limb as his own, particularly in the absence of visual input [39]. It can be divided in a posterior or sensory variant, which relates to sensory hemi-neglect. Hand or arm elevation may be present, which is usually position-dependent and can be induced by sensory stimuli. The patient has a sense that the arm does not belong to him [40]. The anterior or motor variant is characterized by extreme utilization and grasping behavior, more often of the dominant hand, which can result in inter-manual conflict (i.e. one hand unintentionally interfering with or even obstructing purposeful movements of the other hand) [41]. A bilateral, asymmetric alien limb syndrome has been reported [42].

Clinical-pathological studies have proven that many CBD patients can present with other syndromes. These include prominent language disturbances, which more commonly manifest as the non-fluent agrammatic variant of primary progressive aphasia. Some CBD patients exhibit a frontal – behavioral phenotype, which cannot readily be distinguished from the behavioral variant of frontotemporal dementia. However, these patients commonly have additional visuospatial and visuoconstructive deficits, which are rarely encountered in behavioral variant FTD (bv-FTD). Another common phenotype is a Richardson-like syndrome, with prominent supranuclear palsy and early falls, as well as frontal-dysexecutive and pseudobulbar syndrome [4].

Apart from these established clinical phenotypes of CBD, there are rarer manifestations of this disease. These include dementia with an amnestic phenotype (AD-like) [43,44], progressive orofacial apraxia [45], conduction-type aphasia with prominent difficulty in repetition [46], posterior cortical atrophy, with optic ataxia, oculomotor apraxia and simultagnosia [47], frontal-type gait disorder [48] and a prominent pseudobulbar syndrome with dysarthria and emotional lability [49].

## 6 Genetics

CBD is a predominantly sporadic disease. Familial cases due to a microtubule associated tau protein (MAPT) mutation (Ν296Ν) have been reported [50]. The family in this case harbored neuropathological characteristics of CBD but had a clinical phenotype of early-onset dementia with prominent behavioral symptoms. Moreover, the G389R, p.N410H and P301S MAPT mutations have been described in cases of sporadic CBS [[51], [52], [53]].

LRKK2 mutations, classically underlying familial case of Parkinson's disease, can also rarely present as CBS [54,55]. Progranulin (PGRN) mutations can present with variable phenotypes, including CBS [[56], [57], [58], [59]]. In a northern Italian cohort, PGRN mutations were present in 30% of sporadic CBS patients and in 75% of familial CBS patients [60]. Interestingly, this region has an extremely high percentage of familial CBS [61]. The hexanucleotide (GGGGCC) repeat expansion in intron 1 of C9orf72 gene, which is usually associated with FTD-MND spectrum disorders and relates to TDP-43 pathology, has also been reported to present as CBS [62].

A single report of familial 4R-tauopathy has also been reported, with pathologic confirmation of CBD and PSP in two siblings. No mutation was recorded [63].

Analysis of tau polymorphisms in 57 pathologically confirmed CBD patients, revealed a higher frequency of the HQ and H1/H1 haplotype in this cohort, as is the case in PSP patients. No pathogenic mutation in MAPT was evident in any of the patients [64].VEGF haplotypes have also been reported to confer an increased risk for CBS [65]. A large genome-wide association study found significant genetic overlap between CBD and PSP in the MAPT H1 region, as well as SNPs in or near MOBP, CXCR4, EGFR, and GLDC. Interestingly, there was genetic overlap only in the MAPT haplotype between CBD and FTD [66]. A family with a presenilin 1 mutation, AD pathology and CBS phenotype has also been described [67].

## 7 Imaging

### 7.1 Spect – PET studies

Functional imaging modalities have been applied to assist in the differential diagnosis of atypical Parkinsonism, as well as in an attempt to characterize the underlying pathology in a patient with CBS. Perfusion studies using [123I]iodoamphetamine-SPECT indicated that there is decreased perfusion in the inferior prefrontal, sensorimotor, and posterior parietal cortices of CBS patients compared to PSP. Basal ganglia perfusion did not discriminate between the two diseases. CBD patients had more extensive and asymmetric rCBF reductions than in PSP, with the two diseases sharing medial frontal involvement [68]. Likewise, by use of ^99m^Tc HmPaO SPECT, CBD patients could be discriminated from PD patients, since CBD patients exhibited decreased perfusion in the temporoinsular, temporoparietal, and frontal medial regions [69].

A recent FDG-PET study has implied that there are differences in brain perfusion in patients with AD, PSP and CBD who present as CBS. CBS-AD patients presented with posterior, asymmetric hypometabolism, including the lateral parietal and temporal lobes and the posterior cingulate. PSP-CBS had a more anterior hypometabolic pattern, including the medial frontal regions and the anterior cingulate. CBS-CBD showed a similar pattern to CBS-AD, with a more marked, bilateral involvement of the basal ganglia [70].

Another approach is the use of radioligands, which bind to specific pathological proteins, thus providing in vivo information on the disease underlying a patient with CBS. [(11)C]Pittsburgh compound B is strongly indicative of Alzheimer's disease, as it binds to amyloid pathology. By use of this ligand, CBS-AD patients were found to have greater visuospatial and sentence repetition deficits, as well as different cortical atrophy patterns compared to CBS patients with a non-AD pathology [71]. More interestingly, over the past 3 years radioligands have emerged, that bind to tau protein. (18)F-AV-1451 PET provided different binding profiles among CBD, AD and PSP patients, indicating a role in their differential diagnosis. Interestingly, binding of this radioligand correlated with tau pathology burden, but did not correspond to cortical atrophy or hypometabolism in CBD patients [72].

Methodological issues however have arisen, since a mismatch between ante- and postmortem binding to 4R tau lesions was evident. Furthermore, it seems this ligand only binds to a small fraction of 4R pathology [73]. Interestingly, (18)F-AV-1451 binding depended on the presence of amyloid pathology and on the clinical presentation in another study [74]. General issues that have to be resolved relate to non-specific radioligand binding and the specificity of binding to tau isotypes (i.e., 3R vs. 4R vs mixed pathology). Other radiotracers, such as 18F-THK5351 PET have also been tested with the same goal [75].

### 7.2 MRI studies

The imaging hallmark of CBD is the asymmetrical cortical atrophy, which is more pronounced in the peri-rolandic region (anterior and posterior central gyrus), posterior frontal and parietal lobes, contralaterally to the clinically more severely affected side. Cortical atrophy asymmetry becomes more pronounced as the disease progresses.

An abnormally increased signal on proton density MRI sequences in the region of maximal atrophy has also been described in as many as 80% of patients [76]. This could represent demyelination secondary to axonal damage rather than gliosis. Basal ganglia atrophy can also be present, with abnormally increased signal in T2 weighted images, particularly in the posterior lateral border of the putamen [[77], [78], [79]].

Midbrain atrophy, as well as corpus callosum atrophy, more prominent posteriorly can also be evident [80,81]. The “eye of the tiger” sign, considered pathognomonic for pantothenate kinase-associated neurodegeneration, has also been described in a CBD patient [82].

In volumetry studies, CBS patients present cortical atrophy which is more prominent in the posterior frontal lobes (supplementary motor cortex, dorsal premotor and prefrontal cortex, anterior central gyrus) as well as in the anterior parietal lobe (upper parietal lobule). Less severe atrophy is found in the superior temporal and parahippocampal gyri, the caudate, thalamus and cerebellum [83]. Rate of atrophy is greater in the premotor cortex, the primary motor cortex, the somatosensory region 3a, the superior parietal region and the corticospinal tracts. Subcortically, maximum atrophy rate was recorded in the head of the caudate, the putamen, the globus pallidus, the motor region of the thalamus (anterior ventral and lateral ventral nuclei) and substantia nigra [84].

Differences in atrophy patterns in CBS depend on the underlying pathology. Premotor cortex, supplemental motor area and insula are affected irrespective of the pathology and are characteristic of CBS-CBD and CBS-PSP. However TDP-43 patients exhibit more pronounced frontotemporal atrophy (particularly in the prefrontal cortex) and AD patients frontoparietally (particularly parietally) [85]. CBS patients with underlying AD had more prominent posterior temporal and inferior parietal atrophy compared to non-AD pathology, based on volumetry [86].

Diffusion studies indicate that CBD patients exhibit increased mean diffusivity values in the anterior and posterior central gyrus, the middle frontal gyrus bilaterally and the superior and inferior frontal gyrus contralateral to the most affected side [87]. Likewise, by means of diffusion indices, CBS patients exhibited lower fractional anisotropy and greater apparent diffusion coefficient (ADC) values in the corticospinal tract and posterior corpus callosum compared to controls [88]. Diffusion indices may also assist in the differential diagnosis of CBS from PD patients, because CBS patients exhibit abnormal diffusion, particularly in the posterior segments of the corpus callosum [89]. Diffusion tensor imaging may assist in differentiating PSP from CBS patients. CBS showed a more asymmetric, posterior and supratentorial pattern of degeneration, whereas PSP exhibited more infratentorial (particularly midbrain) and symmetric pattern [90]. Furthermore, putaminal ADC values discriminated PD patients from PSP and CBD patients in a separate study. Superior cerebellar peduncle diffusion was more affected on PSP patients compared to CBD [91]. Diverse thalamic involvement in atypical Parkinsonism via diffusion measurements can also assist in differentiating PSP from CBD. PSP patients have more affected anterior and medial thalamic nuclei, whereas in CBD the motor thalamus is more affected [92].

Resting state MRI has also been applied to look into functional connectivity, particularly of thalamic and cerebellar dentate nucleus networks. It was stated that dentate nucleus connectivity differed between PSP and CBS [93].

Another approach applied recently is a multimodal analysis of gray and white matter alterations, by use of volumetry, cortical thickness and diffusion measures. This approach yielded a significant decrease in cortical thickness in the prefrontal cortex, precentral gyrus, supplementary motor area, insula, and temporal pole bilaterally in CBS when compared to controls. Volume loss was evident in the putamen, hippocampus, and accumbens bilaterally as well as the corpus callosum in CBS [94]. When applied in a cohort of CBS and PSP patients, cortical thickness of the peri-rolandic region best discriminated CBS patients from PSP, whereas volumetry was not useful to this end [95].

The same methodology was applied to differentiate CBS-AD from CBS-non AD patients. Diffusion tensor abnormalities were more severe in the corpus callosum, corticospinal tract, and superior longitudinal fasciculus in CBS-non AD, whereas gray matter abnormalities were prominent in the precuneus and posterior cingulate in CBS-AD [96].

## 8 CSF biomarkers

Most studies have reported elevated total CSF tau protein compared to healthy controls [[97], [98], [99], [100], [101], [102]], although this difference did not always reach statistical significance [[103], [104], [105], [106]]. Few studies have reported increased total tau in CBS patients compared to PD [102,105], PSP [98,99,102], PDD [105] and DLB [105]. This has been proposed to represent an inherent biochemical element of CBD. A single study, however, has argued that this tau elevation may in fact represent the inclusion of CBS-AD patients in the CBS cohort. When analyzing the CBS cohort after excluding patients with a typical AD profile (elevated t-tau, ph-tau and decreased Ab42), tau protein did not differ between CBS patients and other patient groups. This emphasizes the need for CSF profiling, with exclusion of patients with typical AD-CSF profile, before examining for between-group differences in CSF biomarkers [107].

Ab42 concentration in CSF does not seem to assist in the differentiation of CBD from patients with atypical Parkinsonism or PD. A single study reported decreased Ab42 levels in CBD [103]. The same goes for τp181, with the exception of few studies which have reported elevation in CBS compared to PD and healthy controls [102] and to MSA [105]. Other studies in the field do not report a difference [101,105,106].

Neurofilament heavy chain was elevated in PSP and MSA patients compared to PD and CBS patients in a single study [108]. Neurofilament light protein also seems to be elevated in atypical Parkinsonism compared to PD, whereas glial fibrillary acidic protein (GFAF) does not seem to differentiate between PD and atypical Parkinsonism. Moreover these proteins' concentrations do not seem to alter over time [109]. This was further supported by a meta-analysis on the subject of NFL in the discrimination of PD from atypical Parkinsonism [110].

Application of an immune-PCR assay for measuring 4R- and 3R-tau isoforms in CSF did not reveal differences between 4R-tauopathies (PSP and CBD) and other causes of atypical Parkinsonism [111].

A novel approach is proteomics analysis through liquid chromatography – mass spectrometry analysis. A single study using this methodology produced several proteins (including acute phase/inflammatory and neuronal/synaptic markers), which could potentially serve as biomarkers in atypical Parkinsonism [112].

## 9 Treatment

No disease-modifying treatment has been approved for CBD. However, many symptoms of CBS can be symptomatically treated. Parkinsonism in CBS can be treated with levodopa, with poor and only transient response [113]. Dystonia in CBS can be successfully treated with botulinum toxin, which can temporarily improve the functionality of the affected limb [114]. Clonazepam is the first choice treatment for myoclonus [113].

Few studies indicate that physical therapy is important in CBS. Physical therapy aims to improve everyday functionality, prevent contractures, and improve rigidity [115]. No treatment has been approved for the cognitive deficits in CBS. However, it is reasonable to try acetylcholinesterase inhibitors in cases of CBS where biomarkers indicative of Alzheimer's disease are available (e.g. CSF biomarkers or [(11)C]Pittsburgh compound B PET-scan).

## 10 Conclusions

CBD can present with a multitude of phenotypes apart from the classical CBS, including behavioral, language or postural deficits. Conversely, CBS can harbor diverse pathologies, including CBD, AD, PSP and FTD-TDP43 among others. This clinical-pathological overlap emphasizes the need for biomarkers to assist in the *ante mortem* etiological diagnosis of a patient with CBS. To this extent, structural and functional imaging modalities, as well as CSF biomarkers have been applied. Further research on biomarkers is critical, in view of specific protein-targeting, disease modifying treatments tested in clinical trials.

## Declaration of competing interest

The authors does not have any conflict of interest.
